# New Insight on the In Vitro Effects of Melatonin in Preserving Human Sperm Quality

**DOI:** 10.3390/ijms23095128

**Published:** 2022-05-04

**Authors:** Sergio Minucci, Massimo Venditti

**Affiliations:** Dipartimento di Medicina Sperimentale, Sez. Fisiologia Umana e Funzioni Biologiche Integrate “F. Bottazzi”, Università degli Studi della Campania “Luigi Vanvitelli”, Via Costantinopoli, 16, 80138 Napoli, Italy

**Keywords:** melatonin, human sperm, oxidative stress, PREP, RSPH6A, DAAM1, PTMA, IAM38, motility, acrosome reaction

## Abstract

Spermatozoa (SPZ) are sensitive to stressful conditions, particularly oxidative stress, which alters their quality; thus, the use of protective molecules as an antioxidant is encouraged. Herein, we used melatonin (MLT) to investigate its in vitro effects on human sperm parameters under conditions of oxidative stress induced by cadmium (Cd). Fifteen human semen samples were divided into control, Cd-treated, MLT-treated, and Cd+MLT-treated groups and analyzed after 30 min, 6 h, and 24 h of exposure. Results showed a time-dependent decrease in SPZ motility, DNA integrity, and increased apoptosis induced by oxidative stress, and these effects were counteracted by MLT co-treatment. Based on these data, we further explored additional parameters just at 24 h. The induced oxidative stress, highlighted by the increased lipid peroxidation, reduced the percentage of SPZ able to undertake acrosome reaction and altered the levels and localization of some protein markers of motility (PREP, RSPH6A), morphology (DAAM1), and acrosome membrane (PTMA, IAM38); all these effects were counteracted by MLT co-treatment. Interestingly, MLT alone was able to ameliorate motility at 30 min of incubation compared to the control, while at 24 h, it prevented the physiological alteration in terms of motility, DNA integrity, and apoptosis. Collectively, the data encourage MLT use as an integrative molecule to ameliorate human gamete quality when compromised by stressful conditions.

## 1. Introduction

Reproduction is a fundamental process that ensures the continuity of a species and genetic variability. This is achieved with the production and differentiation of good quality gametes that must be able to fertilize or be fertilized. Spermatozoa (SPZ), the final product of male gametogenesis, are highly specialized cells that undergo intimate biochemical and morphological changes both during spermatogenesis [1] and their epididymal transit [2]. Thus, the entire process is extremely delicate and subject to “mistakes” that lower sperm quality. Indeed, currently, we are witnessing a progressive decline in the fertility rate worldwide because of the worsening of both male and female gamete quality [3]. The decline in sperm quality may be attributed to several causes: genetic and anatomical abnormalities [4], disturbance of the hormonal status [5], lifestyle [6,7], and environmental stressors [8,9,10,11,12,13,14,15]. In particular, as the release of these substances is continuous, the exposure of humans to them is unfortunately unavoidable and, often, unconscious [16], so their effect could be “silent”, contributing to the generation of male idiopathic infertility, which stands at 40% [17]. On the other hand, the development, and the perfection of assisted reproductive techniques (ART) are of great help to couples who desire to be parents [18,19]. 

One of the principal hazards that SPZ must deal with is the insurgence of oxidative stress, a status in which an unbalance between free radicals (reactive oxygen and nitrogen species, ROS) and antioxidant systems occurs, obviously in favor of the oxidants [20]. On the one hand, redox metabolism regulates numerous physiological functions, such as SPZ capacitation, acrosome reaction (AR), and sperm-oocyte interactions [21,22]; on the other hand, abnormally high levels of free radicals can theoretically damage all cell components and, at the molecular level, lipids and DNA are particularly susceptible [21]. Lipid peroxidation of SPZ causes damage to the cell membrane, impairing its fluidity and, consequently, capacitation and AR [22], as well as mitochondrial membrane, leading to their dysfunction and reduction in ATP synthesis [22]. Moreover, damage to DNA strongly compromises the paternal genomic contribution to the embryo [23,24]. 

Oxidative stress may occur in “normal” SPZ (presence of ROS-generating leukocytes in semen, SPZ mitochondrial metabolism, exposure to exogenous substances) [21] and in those that must be manipulated for ART [21]. So, research focused on the discovery, and the use of molecules that could alleviate the oxidative status, reducing damages in SPZ physiology, are welcomed [25]. Therefore, to prevent oxidative risks that drastically reduce SPZ quality, culture media are routinely supplemented with antioxidants (e.g., ascorbic acid or α-tocopherol) to protect cells by mitigating the harmful effects posed by ROS [26,27,28]. In this regard, the use of melatonin (MLT), thanks to its free radical’s scavenger and antiapoptotic ability [29,30,31,32], has already been investigated. Indeed, many studies reported that MLT reduced the rate of oxidative stress in human SPZ, having beneficial effects on some quality parameters, such as motility, DNA integrity, and lipid peroxidation [33,34,35,36,37,38,39]. 

Herein we added new insight into the use of MLT to preserve human SPZ quality using cadmium (Cd) as an inductor of oxidative stress in vitro. In fact, it is well known that testis is one of the main targets of this heavy metal, and many papers focused on its ability to generate oxidative stress, as well as other damages, compromising the normal spermatogenesis and, ultimately, decreasing sperm quality, both in animal models [40,41] and humans [42,43].

We recently demonstrated the ability of MLT to counteract the harmful consequences produced by Cd in rat testis and SPZ in vivo [44,45]; in this study, we evaluated its efficacy in counteracting the effects of oxidative stress induced by such heavy metal for the first time in human SPZ. With this in mind, in addition to the parameters usually studied (SPZ motility, DNA integrity, and apoptosis), this paper focused on additional aspects that have never been analyzed before, such as the levels and localization of some protein markers that we previously associated with sperm motility (PREP [46] and RSPH6A [47]), morphology (DAAM1 [46]) and acrosome integrity (PTMA and IAM38 [48]). The aim of this paper was to verify if these proteins may be involved in the mechanisms underlying the ameliorative/counteracting effects posed by MLT other than to confirm its ability as an antioxidant molecule to be used in preserving SPZ quality.

## 2. Results

### 2.1. Cd and/or MLT Effects on SPZ Motility

**Figure 1:**
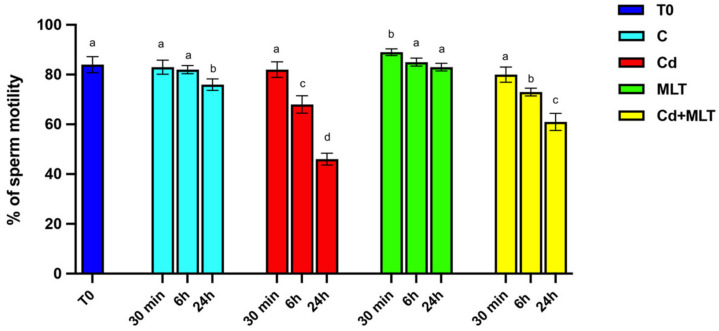
Sperm motility of samples incubated with cadmium (Cd) and/or melatonin (MLT) for 30 min, 6 h, and 24 h. Values are expressed as means ± SEM from 15 samples divided into the 5 groups, including the initial control (T0). a vs. b *p* < 0.05; a vs. c *p* < 0.01; a vs. d *p* < 0.001; b vs. c *p* < 0.05; b vs. d *p* < 0.01; c vs. d *p* < 0.01.

The effects of Cd and/or MLT treatment on sperm motility are shown in Figure 1. 

Interestingly, a slight decrease in SPZ motility was observed only in the 24 h control group as compared to the initial control (T0; *p* < 0.05), suggesting that motility decreases with time, independently of any treatment. 

Data evidenced that Cd produced a time-dependent reduction in SPZ motility. Indeed, while no statistically significant effects on sperm motility were seen at 30 min of incubation with Cd as compared to both T0 and the control, a significant decrease was observed in the group of SPZ Cd-treated at 6 h when compared to the controls (*p* < 0.01). Finally, a consistent decrease in sperm motility in the 24 h Cd group as compared to T0 (*p* < 0.001) and the 24 h control (*p* < 0.01) was observed. The counteracting action of MLT on the effects exerted by Cd on SPZ motility was evidenced at 6 h of treatment, as a significative slight decrease was observed in the Cd+MLT treated group when compared to the control (*p* < 0.05), and it was further confirmed at 24 h of treatment, where the decrease in SPZ motility was less evident than in the Cd group (*p* < 0.01), but it still remained higher than the control (*p* < 0.05). Thus, the reduction in SPZ motility observed in the Cd+MLT group at 24 h was comparable to that observed in the Cd group just after 6 h of treatment (Figure 1). 

Worthy of note are the data coming from the observation of SPZ motility exposed to MLT alone. Indeed, at 30 min of treatment, MLT induced an increase in SPZ motility, as compared to the T0 and control groups (*p* < 0.05). The ameliorative action also persisted at 6 h and 24 h, although no statistical differences were observed between the T0, control, and MLT-treated groups exposed for 6 h, while, at 24 h, the SPZ treated with MLT alone showed higher motility than the time control (*p* < 0.05) and not significantly different from the T0 group. 

### 2.2. Cd and/or MLT Effects on SPZ DNA Integrity and Apoptosis

**Figure 2:**
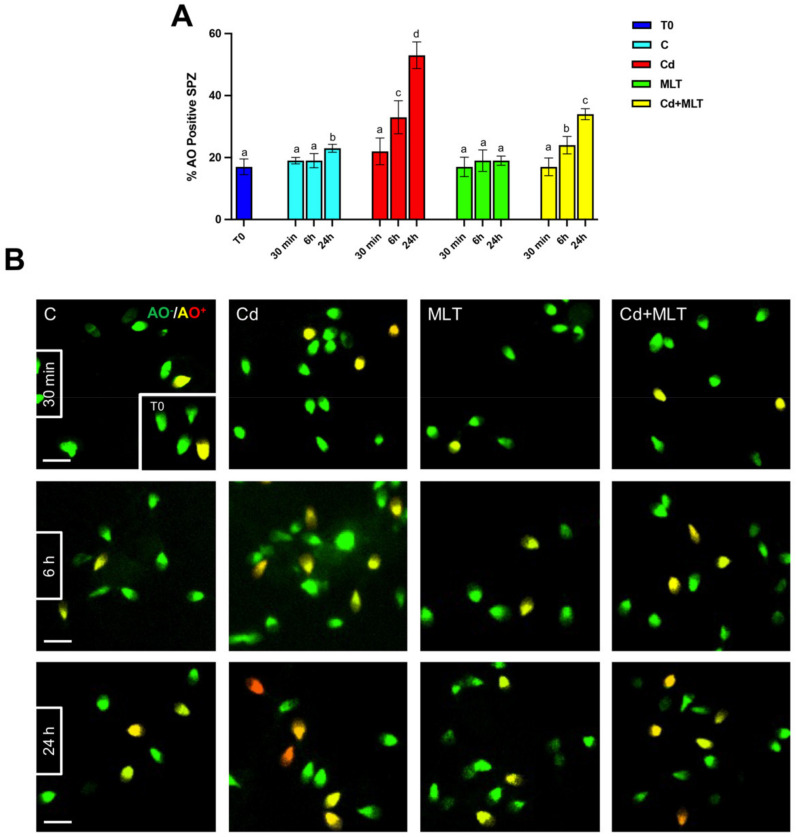
Determination of DNA integrity through the acridine orange (AO) staining of sperm samples incubated with Cd and/or MLT for 30 min, 6 h, and 24 h. (**A**) Percentage of positive AO cells. Values are expressed as means ± SEM from 15 samples divided into the five groups, including the initial control (T0). a vs. b *p* < 0.05; a vs. c *p* < 0.01; a vs. d *p* < 0.001; a vs. d *p* < 0.01; b vs. c *p* < 0.05; b vs. d *p* < 0.01; b vs. e *p* < 0.05; c vs. d *p* < 0.01; c vs. e *p* < 0.01; d vs. e *p* < 0.05. (**B**) AO staining, that highlight the SPZ with damaged DNA (yellow/orange) respect to those with intact DNA (green) in each analyzed time-points. Scale bars represent 10 µm.

**Figure 3:**
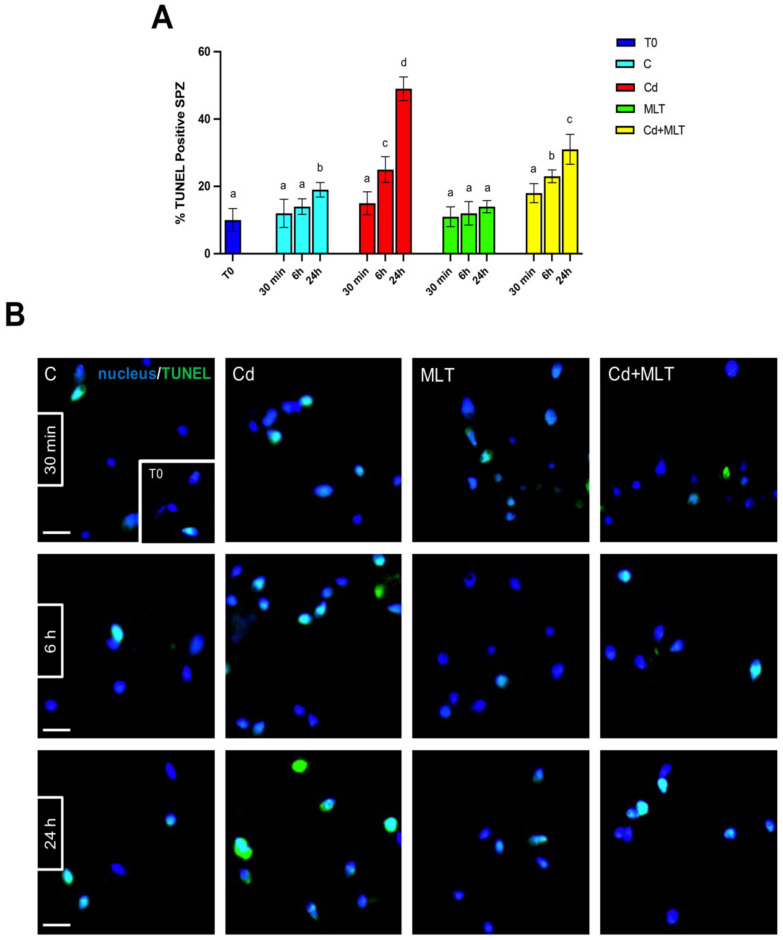
Determination of apoptosis through the TUNEL assay of sperm samples incubated with Cd and/or MLT for 30 min, 6 h, and 24 h. (**A**) Percentage of positive TUNEL cells. Values are expressed as means ± SEM from 15 samples divided into the 5 groups, including the initial control (T0). a vs. b *p* < 0.05; a vs. c *p* < 0.01; a vs. d *p* < 0.001; b vs. c *p* < 0.05; b vs. d *p* < 0.01; c vs. d *p* < 0.01. (**B**) TUNEL assay, that highlight the apoptotic SPZ (green) respect to non-apoptotic (blue) in each analyzed time-points. Slides were counterstained with DAPI-fluorescent nuclear staining (blue). Scale bars represent 10 µm.

The analysis of SPZ DNA integrity and apoptosis, performed with acridine orange (AO) and TUNEL experiments, are shown in Figure 2 and Figure 3, respectively. 

Interestingly, a slight increase in the percentage of both AO and TUNEL positive cells was observed only in the 24 h control group compared to T0 (*p* < 0.05), suggesting that SPZ quality decreases with time, independently of any treatment. 

As observed above, data evidenced that Cd produced a time-dependent increase in the number of SPZ showing damaged DNA and/or apoptosis. Indeed, both AO (Figure 2) and TUNEL (Figure 3) experiments highlighted that the Cd effects were more pronounced after 24 h of treatment. In particular, at 30 min of incubation, neither the percentage of AO (Figure 2A) nor that of TUNEL (Figure 3A) positive cells were affected by the Cd treatment, in which scattered positive cells could be seen (Figure 2B and Figure 3B). At 6 h of incubation, the Cd treatment produced an increase in AO (Figure 2A) and TUNEL (Figure 3A) positive cells as compared to the controls (*p* < 0.05). 

The most consistent data came from the observations at 24 h of treatment; in fact, at this time-point a strong increase in AO (Figure 3A) and TUNEL (Figure 3A) positive cells was evidenced in the Cd-treated group as compared to the T0 (*p* < 0.0001) and to the 24 h control (*p* < 0.001) groups. In addition, the higher degree of DNA damage was also highlighted by the presence of many SPZ showing an orange/red fluorescent signal (Figure 2B). 

The counteracting action of MLT on the increase in SPZ DNA damages and apoptosis as a consequence of Cd treatment was evidenced at 6 h of treatment, and a significative slight increase was also observed in the Cd+MLT treated group as compared to the control (*p* < 0.05), and this action was confirmed at 24 h of treatment, where the percentage of AO and TUNEL positive cells was less pronounced than in that observed in the Cd group control (*p* < 0.01) but still higher than in the control (*p* < 0.05).

Noticeably, at any of the time-points of the treatment, no differences were observed in the percentage of AO and TUNEL positive cells between the SPZ treated with MLT alone and T0 groups, confirming that MLT can prevent the DNA damage and apoptosis of SPZ from an action exerted by MLT. 

### 2.3. Cd and/or MLT Effects on Oxidative Stress

From the analysis of the above combined data, it is possible to observe that the most pronounced effects induced by Cd and the protection exerted by MLT occur at 24 h of exposure. Thus, due to this fact, we chose the 24 h time-point to perform all the subsequent examinations.

**Figure 4:**
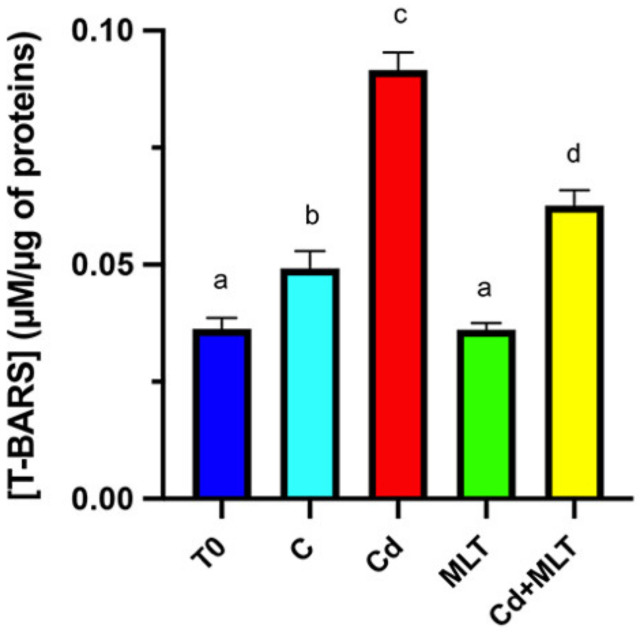
Lipid peroxidation rate evaluated by thiobarbituric acid-reactive species (TBARS) assay of sperm samples incubated with Cd and/or MLT for 24 h. Values are expressed as means ± SEM from 15 samples divided into the 5 groups, including the initial control (T0). a vs. b *p* < 0.001; a vs. c *p* < 0.05; a vs. d *p* < 0.01; b vs. c *p* < 0.001; c vs. d *p* < 0.01.

First, oxidative stress was analyzed using the thiobarbituric acid-reactive species (TBARS) assay, which measures the lipid peroxidation rate of cell membranes (Figure 4). 

The data showed a slight increase in TBARS level in the control group compared to T0 (*p* < 0.05). In addition, Cd exposure provoked a significant increase in TBARS levels when compared to the value of T0 and control SPZ (*p* < 0.001). Conversely, MLT given in combination with Cd partially counteracted the increase in TBARS levels observed in the SPZ of the Cd group (*p* < 0.01) but without reaching the control values (*p* < 0.05). Remarkably, SPZ treated with MLT alone showed decreased TBARS levels compared to those of the control (*p* < 0.05) and was not significantly different from the SPZ of the T0 group.

### 2.4. Cd and/or MLT Effects on PREP, RSPH6A and DAAM1

**Figure 5:**
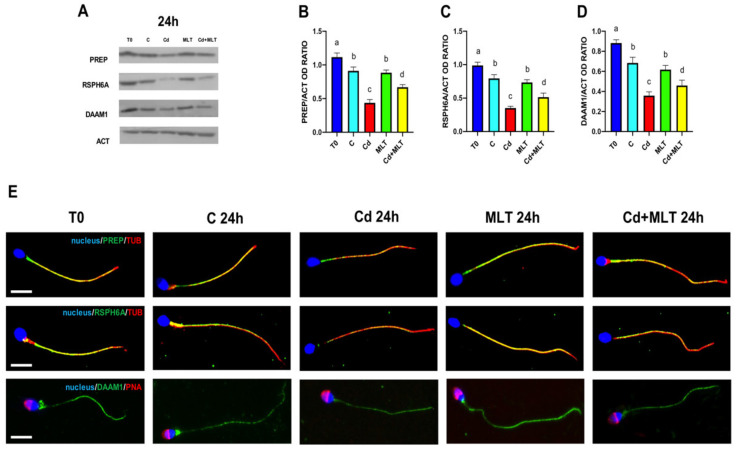
Western Blot (WB) and immunofluorescence (IF) analysis of PREP, RSPH6A, and DAAM1 of sperm samples incubated with Cd and/or MLT for 24 h. (**A**): WB analysis showing PREP (80 kDa), RSPH6A (80 kDa), DAAM1 (112 kDa) and β-actin (42 kDa) protein levels in SPZ treated with Cd and/or MLT. (**B**–**D**): Histograms showing the relative protein levels of PREP (**B**), RSPH6A (**C**), and DAAM1 (**D**), respectively. Data were normalized with β-actin and reported as OD ratio. Values are expressed as means ± SEM from 15 samples divided into the five groups, including the initial control (T0). A vs. b *p* < 0.05; a vs. c *p* < 0.001; a vs. d *p* < 0.01; b vs. c *p* < 0.01; b vs. d *p* < 0.05; c vs. d *p* < 0.01. (**E**) IF analysis of PREP (green, upper panel), RSPH6A (green, middle panel), and DAAM1 (green, lower panel) in SPZ treated with Cd and/or MLT for 24 h. Red represents α-tubulin in the upper and middle panels, and acrosome, marked with PNA lectin, in the lower panel. Slides were counterstained with DAPI-fluorescent nuclear staining (blue). Scale bars represent 10 μm.

To better characterize the protective action of MLT on the adverse effects induced by Cd treatment, we analyzed, for the first time in in vitro treated human SPZ, the levels and the localization of some proteins that in our previous works have been characterized in rat/mouse testis and SPZ, as well as in human sperm: PREP, RSPH6A, and DAAM1 (Figure 5).

Data showed that PREP (Figure 5A,B), RSPH6A (Figure 5A,C), and DAAM1 (Figure 5A,D) protein levels of the control and MLT groups decreased after 24 h of incubation as compared to T0 (*p* < 0.05). The Cd treatment produced a marked decrease in the levels (Figure 5A–D) of all the three analyzed proteins as compared to the control and MLT groups (*p* < 0.01). Contrarily, in the Cd+MLT treated SPZ, MLT partially counteracted the decreased PREP, RSPH6A, and DAAM1 levels since they were higher compared to the Cd group (*p* < 0.01), but they still remained lower than those of the control and MLT groups (*p* < 0.05). 

Immunofluorescence (IF) analysis confirmed the above results (Figure 5E). Indeed, both PREP (upper panel) and RSPH6A (middle panel) signals (in green) were clearly detected in SPZ tails in all the analyzed groups, in co-localization with tubulin (in red), as highlighted by the intermediate yellow/orange tint. In the T0, control, and MLT groups, a comparable distribution pattern and fluorescence intensity were evidenced, while in the Cd-treated SPZ, PREP, and RSHP6A signals were less intense, as can be seen by the predominance of a red signal. In the SPZ co-treated with both Cd+MLT, more intense green signals for both proteins were appreciable due to the partially counteractive action of MLT on Cd effects.

Concerning DAAM1 (Figure 5E, lower panel), the IF results showed that its signal (in green) was mainly detectable inside the flagellum other than in the mid-piece/cytoplasmic droplet of all the SPZ in the analyzed groups. However, in the SPZ exposed to Cd, a decreased green signal intensity was observed, particularly in the mid-piece, where it almost disappeared. Once again, in the Cd+MLT treated SPZ, the DAAM1 distribution and signal intensity were more intense due to the partially counteractive action of MLT on Cd effects.

### 2.5. Cd and/or MLT Effects on Acrosome Reaction, PTMA and IAM38

**Figure 6:**
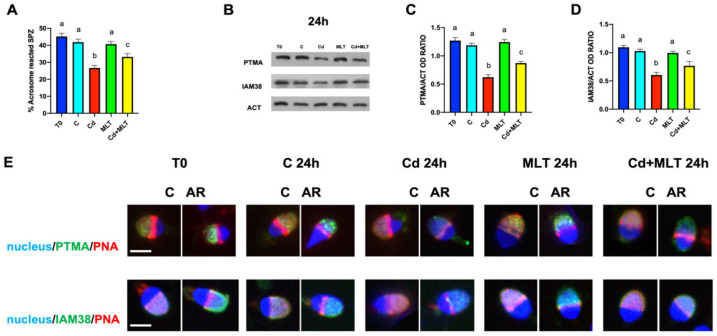
Acrosome reaction, WB, and IF analysis of PTMA and IAM38 of sperm samples incubated with Cd and/or MLT for 24 h. (**A**): Histogram showing the percentage of acrosome reacted SPZ induced by progesterone. (**B**): WB analysis showing PTMA (15 kDa), IAM38 (38 kDa), and β-actin (42 kDa) protein levels in SPZ treated with Cd and/or MLT. (**C**,**D**): Histograms showing the relative protein levels of PTMA (**C**) and IAM38 (**D**), respectively. Data were normalized with β-actin and reported as OD ratio. Values are expressed as means ± SEM from 15 samples divided into the five groups, including the initial control (T0). a vs. b *p* < 0.001 a vs. c *p* < 0.05; b vs. c *p* < 0.01. (**E**): IF analysis of PTMA (green, upper panel) and IAM38 (green, lower panel) in SPZ treated with Cd and/or MLT for 24 h. Red represents α-tubulin in the upper and middle panels, and acrosome, marked with PNA lectin, in the lower panel. Slides were counterstained with DAPI-fluorescent nuclear staining (blue). Scale bars represent 5 μm.

The MLT counteractive action against the effects induced in the SPZ exposed to Cd was further studied by analyzing the in vitro induced acrosome reaction (AR) and the level and localization of two proteins, PTMA and IAM38, notoriously associated with the acrosome membrane (Figure 6). 

Firstly, the data showed no differences in the percentage of SPZ able to perform AR when stimulated by progesterone between the T0, control, and MLT groups (Figure 6A). Contrarily, in the SPZ exposed to Cd, we observed a significant reduction in the ability to perform AR as compared to those observed in the control and MLT groups (*p* < 0.001). In the Cd+MLT treated SPZ, the percentage of reacted SPZ was higher than those observed in the Cd group (*p* < 0.01), but it still remained lower than those of the control and MLT groups (*p* < 0.05).

Secondly, Western blot (WB) analysis showed that in the Cd-treated SPZ, there was an observable decrease in the protein level of both PTMA (Figure 6B,C) and IAM38 (Figure 6B,D) as compared to the T0, control, and MLT groups (*p* < 0.01) In the Cd+MLT SPZ, the PTMA and IAM38 protein levels were significantly lower than control and MLT groups (*p* < 0.05) but higher than the Cd group (*p* < 0.01).

Thirdly, to investigate whether Cd and/or MLT could modify PTMA and IAM38 distribution after the membrane fusion and the exocytosis of the acrosomal soluble content, an IF analysis was performed on both “intact” SPZ (C; Figure 6E) and SPZ, whose AR was induced by progesterone (AR; Figure 6E) in all the considered groups. 

In all the groups, both PTMA and IAM3 localization was evident in the upper part of the SPZ head, corresponding to the acrosomal region. However, in intact SPZ, the green PTMA and IAM38 signals were partially “masked” by the red PNA signal. The occurrence of AR was demonstrated by PNA fluorescence, as the staining decreased in the apical region of reacted SPZ and progressively re-localized to the equatorial region (Figure 6E). PTMA and IAM38 became more exposed, and the signals became more distinct; however, in the SPZ Cd-treated group a decrease in the signal intensity was evident. In the SPZ co-treated with both Cd+MLT, more intense green signals for both proteins were appreciable, due to the partially counteractive action of MLT on Cd effects.

An alternative version of all the figures, prepared with a colorblind palette for sight-challenged or colorblind people, are available in the Supplementary Materials.

## 3. Discussion

It is widely known that male fertility depends on a definite amount of SPZ with sufficiently high quality in terms of concentration, morphology, motility, and DNA integrity [3]. However, a worrying worldwide decrease in fertility, directly proportional to increased deterioration of gamete quality, is occurring [3]. SPZ are extremely specialized cells, able to go through the female genital tract, perform an appropriate AR, and fertilize the oocyte, but, at the same time, they are particularly delicate and sensitive to most kinds of physical, chemical, and biological stressors [49]. Between them, ROS-generating oxidative stress seems to influence SPZ physiology more than others for two reasons: (1) the scarcity of appropriate antioxidant defenses due to their exiguous cytoplasm, and (2) the presence of high concentrations of polyunsaturated fatty acids in their plasma membrane, which can be easily oxidated altering its fluidity [20]. The source of ROS may be either endogenous (SPZ metabolic activity, presence of leukocytes in semen) [20] or exogenous, occurring during the SPZ sample preparation procedures for ART [50]. Indeed, SPZ become particularly exposed to ROS when seminal plasma is removed and/or cryopreserving agents are added to samples; for this, the development of increasingly sophisticated and successful ART is helpful to the outcome of pregnancy [50]. In this regard, many efforts have been made to develop methods, such as the addition of antioxidant molecules to cryopreserving agents, with the aim of achieving the best environment to preserve SPZ quality as much as possible [26,27,51]. Among these, MLT is one of the most studied molecules due to its widely acknowledged antioxidant and antiapoptotic properties [29,30,31,32,33,34,35,36,37,38,39]. 

In this paper, we further expanded the information concerning the protective action of MLT when ROS generation is favored, using Cd as a pro-oxidative agent in human SPZ in vitro. The choice of Cd was not casual, as its ability to generate oxidative stress is well-known [52], and we recently demonstrated its deleterious effects in the rat testis and SPZ in vivo [44,45,53,54]. 

Firstly, our data showed that, although the sperm motility, DNA integrity, and viability were preserved after 30 min and 6 h of incubation, at 24 h they were slightly altered as compared to the initial values. This result suggested that SPZ quality decreased regardless of any treatment, further recommending the use of preserving agents to keep the integrity of these parameters. Secondly, results indicated that, when ROS generation was accelerated by Cd, in a time-dependent manner, the motility of SPZ considerably decreased, while DNA damages and the apoptotic rate increased. However, when MLT was given in combination with Cd, data indicated a partial counteraction to the harmful effect provoked by Cd, confirming the ability of MLT to reduce the effects produced by the oxidative milieu in human SPZ in vitro. 

Cd effects were not surprising, as its consequences on spermatogenesis are well recognized [55]; however, the precise mechanism of action is still poorly understood, and it is not excluded that it may be due to multiple causes, independent from each other. One of the most accepted theories is just the ability of Cd to induce oxidative stress, which is responsible for many impairments in SPZ physiology. 

Although MLT has a potent antioxidant property, its effect on motility was only partial as it was not able to counteract the effect of Cd completely, and this may be due to the involvement of other underlying mechanisms. Indeed, regarding motility (as well as other processes, as discussed below), intracellular calcium (Ca^2+^) concentration represents a fundamental regulatory factor, and, due to the scarce SPZ cytoplasm and, consequently, the weakly developed Ca^2+^ storage, its influx from the external environment is the predominant source of this element for the normal SPZ physiology [56]. It has been suggested that the sperm-specific cation channel (CatSper) acts as the main channel for Ca^2+^ influx and that its permeability is interfered with by Cd exposure [57]. Thus, we hypothesized that the partial MLT protection might be due to its inability to prevent/contrast the effect of Cd on CatSper permeability to Ca^2+^, but just on reducing oxidative stress. 

Worthy of note is the data coming from the treatment with MLT alone; in fact, at 30 min of incubation, an increase in SPZ motility was observed compared to the control, while at 24 h, it prevented the physiological alteration in terms of motility, DNA integrity, and apoptosis as observed in the time-point control group. This may be an effect of MLT either stimulating the mitochondrial activity, enhancing the efficiency of the membrane electron transport chain and, consequently, ATP synthesis [58], or reducing the expression of proapoptotic proteins (caspase 3 and 9), increasing SPZ viability [34,59].

The combined data coming from the timed experiments indicated that 24 h represents the time-point showing the most pronounced effects; for this (and also considering the large amount of sample required for the other examinations), we decided to complete the study of all the additional parameters just at this time-point. 

The first evaluated parameter was the oxidative stress status via the TBARS assay, which measures the rate of lipid peroxidation. Not surprisingly, an increased TBARS concentration was observed in the SPZ exposed to Cd, confirming the insurgence of oxidative stress. However, the slight increase observed also in the SPZ of the control group evidenced that little oxidative damages happened at 24 h, independent of any kind of treatment; this, in turn, highlighted, once again, the need for molecule(s) to be able to preserve SPZ physiology. The use of MLT, given alone or in combination with Cd, confirmed its ability to reduce oxidative stress, further evidencing that it may be a good choice for such a purpose.

A proper organization of cytoskeletal elements and of its numerous associated proteins is strictly correlated to a normal SPZ morphology and motility. We analyzed MLT’s counteractive action on the oxidative stress induced by Cd on the levels and localization of three proteins involved in the cytoskeletal dynamics that we previously correlated to SPZ physiology: PREP [46,60], RSPH6A [47], and DAAM1 [46,61]. 

PREP is a serine protease that has been associated with the C-terminus of α-tubulin, suggesting its involvement in microtubule-related processes, independent of the enzymatic activity [62]. We have also demonstrated that PREP-/- mice presented reduced sperm motility [60], as well as the association of asthenospermia with impairment of PREP protein level and localization in human SPZ [46]. RSPH6A is a testis-specific protein that localizes specifically in the tail of mature SPZ; we demonstrated that the expression and localization of RSPH6A in SPZ samples coming from patients affected by myotonic dystrophy type 1, a degenerative pathology of the muscular tissue associated to reproductive disorders, was lower as compared to those of normosperm [47]. 

Present results indicated, for the first time in human SPZ, that the protein level of PREP and RSPH6A were reduced by Cd-induced oxidative stress and that such a reduction was counteracted by MLT. So, concerning SPZ motility, in addition to the above-mentioned effects, we could also hypothesize that the reduction in PREP and RSPH6A levels, other than their altered localization, may contribute to the impaired motility observed in the Cd group.

Finally, DAAM1 belongs to the formin protein family, regulating the polymerization of actin microfilaments [63]. In normal human SPZ, we previously found that DAAM1 localized in the cytoplasmic droplet, the residual cytoplasm that is phagocyted by Sertoli cells prior to SPZ release in the tubules’ lumen [61]. Contrarily, altered DAAM1 protein level and localization were evidenced in the SPZ samples of terathospermic men, implying its involvement in the differentiation of gametes with a proper morphology [46]. Interestingly, here it was evidenced that the most pronounced effects of Cd on DAAM1 were on its almost complete disappearance in the cytoplasmic droplet. Although such a structure is usually absorbed by Sertoli cells prior to SPZ spermiation, at the same time, it has been proposed to be an important sperm-quality parameter [64], as it seems to regulate the SPZ volume in hypo- and hyperosmotic environments, giving them resistance to hostile environments, such as the migration through cervical mucus [65]. Thus, the lack of DAAM1 in the cytoplasmic droplet may also disturb SPZ in the way towards the oocyte.

Furthermore, MLT was effective in counteracting the decrease in DAAM1 protein levels, probably induced by oxidative stress, and consequently preserving its localization in the cytoplasmic droplet, contributing to maintaining SPZ quality. 

The last parameter here considered is the ability of SPZ to complete the AR, the mechanism during which the acrosome membrane fuses with the plasma membrane under the stimulus of Ca**^2+^** influx, leading to the release of the soluble content of the acrosome [66]. In this scenario, the integrity of membrane components, lipids, and protein is fundamental. Considering that these components are two main targets of oxidative stress, we analyzed the percentage of SPZ able to perform AR after progesterone stimulation, as well as the protein levels and localization of two markers of the inner acrosomal membrane: PTMA and IAM38. In our previous works, PTMA, one of the most acidic polypeptides of mammals, was associated with the post-meiotic progression of germ cells [48,67,68]; moreover, the role of PTMA in the interaction of SPZ with the oocyte and in the male pronuclear chromatin decondensation was also hypothesized [48]. IAM38 is a receptor of the inner acrosome membrane, mediating the ‘‘secondary binding’’ of the SPZ to oocyte and its subsequent penetration of the zona pellucida [69].

The present data clearly showed that the exposure to Cd reduced the ability of SPZ to perform a proper AR. This may be due to altered membrane fluidity because of the oxidative status, producing the increased lipid peroxidation rate, as also highlighted by the TBARS assay. Moreover, the lower percentage of reacted SPZ could also be due, as above-mentioned, to the reduced entry of Ca**^2+^**, induced by progesterone, via the CatSPer channel.

Additionally, following the decreased protein levels of PTMA e IAM38, their localization signal in the SPZ head was less intense. Thus, the alterations produced by Cd on SPZ’s ability to perform a proper AR, as well as on these two markers of the inner acrosomal membrane, suggesting that the oxidative stress may also interfere with SPZ-oocyte interaction during fertilization events.

Once again, the contemporaneous administration of MLT reduced all the above effects; indeed, the % of reacted SPZ and PTMA and IAM38 protein levels and localization was similar to those of the control.

## 4. Materials and Methods

### 4.1. Human Semen Samples, Exposure Procedure, In Vitro AR and Ethical Approval

Semen samples were obtained by masturbation from 15 nonsmoking patients between 27 and 35 years and left to liquefy at 37 °C for 30 min. Semen analysis was carried out following WHO guidelines [70] (see Table S1). For all the subsequent analyses, motile sperm were selected by the direct swim-up procedure [48]. All the samples had a progressive motility of >90%. Each purified sample was divided into 5 aliquots (2 × 10^6^ sperm/mL): (1) initial control (T0); (2) experimental control (C); (3) Cd-treated (10 μM CdCl_2_; Sigma-Aldrich; Milan, Italy) [43]; (4) MLT-treated (2 mM MLT, Sigma-Aldrich; Milan, Italy) [71]; (5) Cd+MLT-treated (10 μM CdCl_2_ + 2 mM MLT). The incubation was performed at 37 °C for 30 min, 6 h, and 24 h. Fresh medium prepared as described above, was added after 8 h of incubation up to the point of 24 h.

Moreover, after 22 h of exposure, an aliquot from all the groups was taken, and 15 μM of progesterone in ethanol was added to induce the acrosome reaction, at 37 °C for 2 h. At the end of each time point and treatment, an aliquot (500 μL) was taken from each sample, centrifuged for 5 min at 1500 rpm, the pellet resuspended of PBS, and motility was evaluated. Finally, aliquots for all the time-points were spotted and air-dried on slides and stored at −20 °C, while, for T0 and 24 h time-points, the remaining sample was centrifuged again, the supernatant removed, and the resulting pellet stored at −80 °C. 

Patients were informed about the purpose of the study and written informed consent for the use of the semen remaining after the routine analysis was obtained. The study was conducted according to the guidelines of the Declaration of Helsinki and approved by the Ethics Committee of “Università degli Studi della Campania Luigi Vanvitelli” (protocol code 206 approved on 15 April 2019).

### 4.2. DNA Integrity and Apoptosis Assessment

Acridine orange (AO) staining was employed on formalin-fixed SPZ to evaluate the DNA integrity rate, following Tejada et al. [72]. Apoptosis was examined in formalin-fixed SPZ by the TUNEL-assay using the DeadEnd™ Fluorometric TUNEL System (#G3250; Promega Corp., Madison, WI, USA) following the manufacturer’s protocol. The cell nuclei were marked with DAPI (#MBD0015; Sigma–Aldrich, Milan, Italy).

All the slides were observed under a fluorescent microscope with a UV lamp (Leica DM 5000 B+CTR 5000; Leica Microsystems, Wetzlar, Germany) and saved using the IM 1000 software (version 4.7.0; Leica Microsystems, Wetzlar, Germany). Photographs were taken using the Leica DFC320 R2 digital camera. A total of about 300 SPZ/slide was counted, and the parameter was expressed as a percentage of yellow/orange/red SPZ (positive AO) or as a percentage of TUNEL positive SPZ.

### 4.3. TBARS Assay

SPZ lysate (see point 4.4) were processed as described in Venditti et al. [73]. Briefly, samples were incubated with 0.5 mL of 0.78% aqueous solution of thiobarbituric acid and 0.5 mL of 20% acetic acid with a final pH of 3.5. Samples were then heated for 45 min at 95 °C and subsequently centrifuged at 700 rpm. for 5 min. Supernatants were collected, and TBARS were quantified by spectrophotometry at 532 nm. Results were expressed as TBARS μM/μg of protein extract. Each measurement was performed in triplicate.

### 4.4. Protein Extraction and WB Analysis

Total proteins were extracted using RIPA lysis buffer (#TCL131; Hi Media Laboratories GmbH; Einhausen, Germany) supplemented with 10 μL/mL of protease inhibitors mix (#39102; SERVA Electrophoresis GmbH; Heidelberg, Germany). The protocol of WB was consistent with our previous study [74,75]. The primary and secondary antibodies were used as follows: (1) anti-PREP (1:2000; #ab58988; Abcam, Cambridge, UK); (2) anti-RSPH6A (1:1000; #HPA045382; Sigma–Aldrich, Milan, Italy); (3) anti-DAAM1 (1:1000; #E-AB-13182, Elabscience Biotechnology, Wuhan, China); (4) anti-PTMA (1:1000; #ab247074; Abcam, Cambridge, UK); (5) anti-IAM38 (#ab97691; Abcam, Cambridge, UK) and (6) anti-β-Actin (1:5000; #E-AB-20031; Elabscience Biotechnology, Wuhan, China) (7) goat anti-mouse IgG (#AP130P; Sigma-Aldrich, Milan, Italy) for the mouse anti- β-Actin, (8) goat anti-rabbit IgG (#AP307P; Sigma-Aldrich, Milan, Italy) for all the others, both diluted 1:10,000. ImageJ software (version 1.53 g) was used to analyze all bands. All the WB were performed in triplicate.

### 4.5. IF Analysis

The SPZ were fixed in 4% paraformaldehyde in PBS for 10 min at RT and then in PBS and incubated with 0.1% Triton X-100 (#T8787; Sigma-Aldrich, Milan, Italy) in PBS [76]. The slides were blocked with a PBS solution containing 20% of normal goat serum (#NS02L Sigma-Aldrich, Milan, Italy) and 5% BSA (#05470; Sigma-Aldrich, Milan, Italy) before the addition of the abovementioned primary antibodies and anti-α-tubulin antibody (#E-AB-20036; Elabscience Biotechnology, Wuhan, China) all were diluted to 1:100 and incubated overnight at 4 °C. After washing with PBS, the slides were incubated for 1 h with PNA lectin (#L32458; Thermo Fisher Scientific, Waltham, MA, USA) diluted at 1:50, and the appropriate secondary antibody [goat anti-rabbit Alexa Fluor 488, (#A32731 Thermo Fisher Scientific, Waltham, MA, USA); goat anti-mouse CF™ 568 (#SAB4600082; Sigma–Aldrich, Milan, Italy)] both diluted to 1:500 in the blocking mixture. In all IF analyses, the slides were mounted with DAPI for nuclear staining and observed under a fluorescent microscope.

### 4.6. Statistical Analysis

Data are shown as mean ± standard error of the mean (SEM). Statistical analysis was performed by using one-way ANOVA followed by a Tukey post hoc t-test performed with Prism 5.0, GraphPad Software (San Diego, CA, USA). Differences between the groups were considered statistically significant at *p* < 0.05.

## 5. Conclusions

Our data further confirmed that oxidative stress damages, produced by Cd exposure, strongly decrease human SPZ quality in terms of motility, viability, DNA integrity, and the ability to perform AR. Moreover, we hypothesized that the alteration in these parameters is accompanied by an impaired protein level and localization of some cytoskeletal (PREP, RSPH6A, and DAAM1) and acrosome membrane integrity (PTMA and IAM38) markers. Furthermore, new insights into the effects of MLT in protecting SPZ against oxidative stress have been added, confirming that MLT is a convenient molecule to be used as an additive to preserve SPZ quality generally or when semen samples are handled for ART.

## Data Availability

The authors confirm that the data supporting the findings of this study are available within the article.

## Supplementary Materials

The following supporting information can be downloaded at: https://www.mdpi.com/article/10.3390/ijms23095128/s1.
