# Supplementary figures and images for: New Insight on the In Vitro Effects of Melatonin in Preserving Human Sperm Quality
Source: Int J Mol Sci. 2022 May 4;23(9):5128. doi: 10.3390/ijms23095128 (PMC9100642; doi:10.3390/ijms23095128)

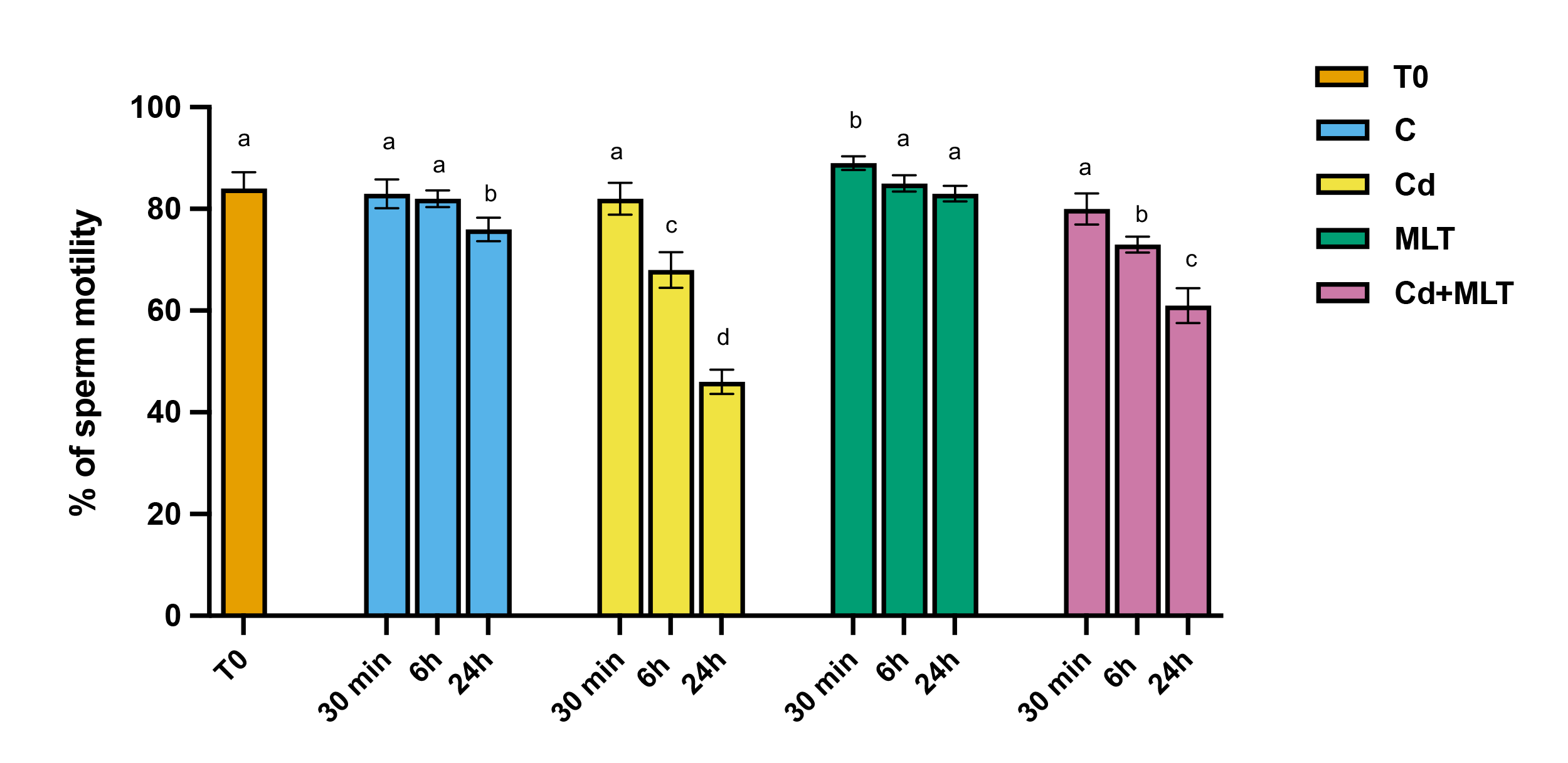

Supplement: Supplementary file 1 [file ijms-23-05128-s001.zip › Figure 1.tif]

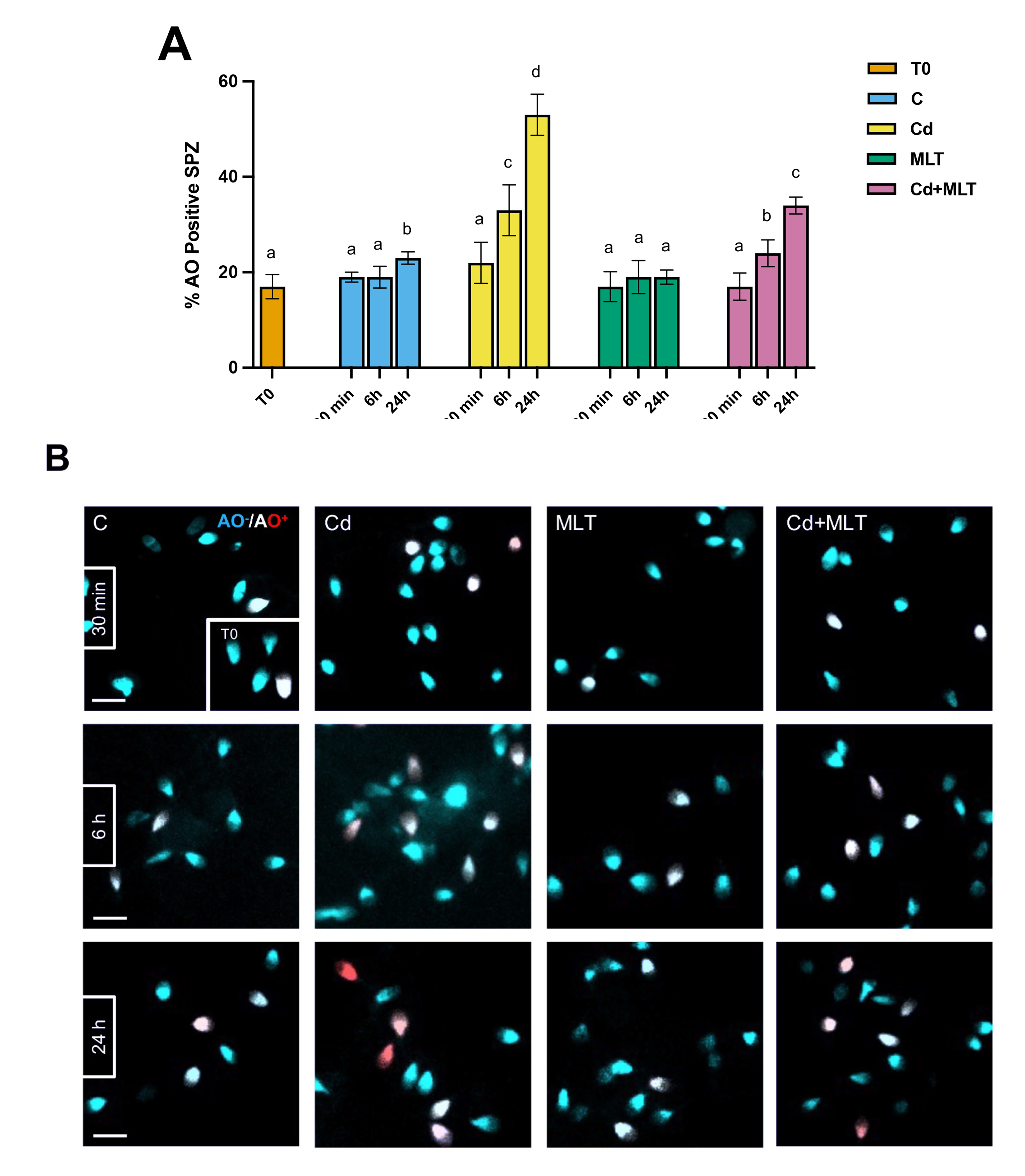

Supplement: Supplementary file 1 [file ijms-23-05128-s001.zip › Figure 2.tif]

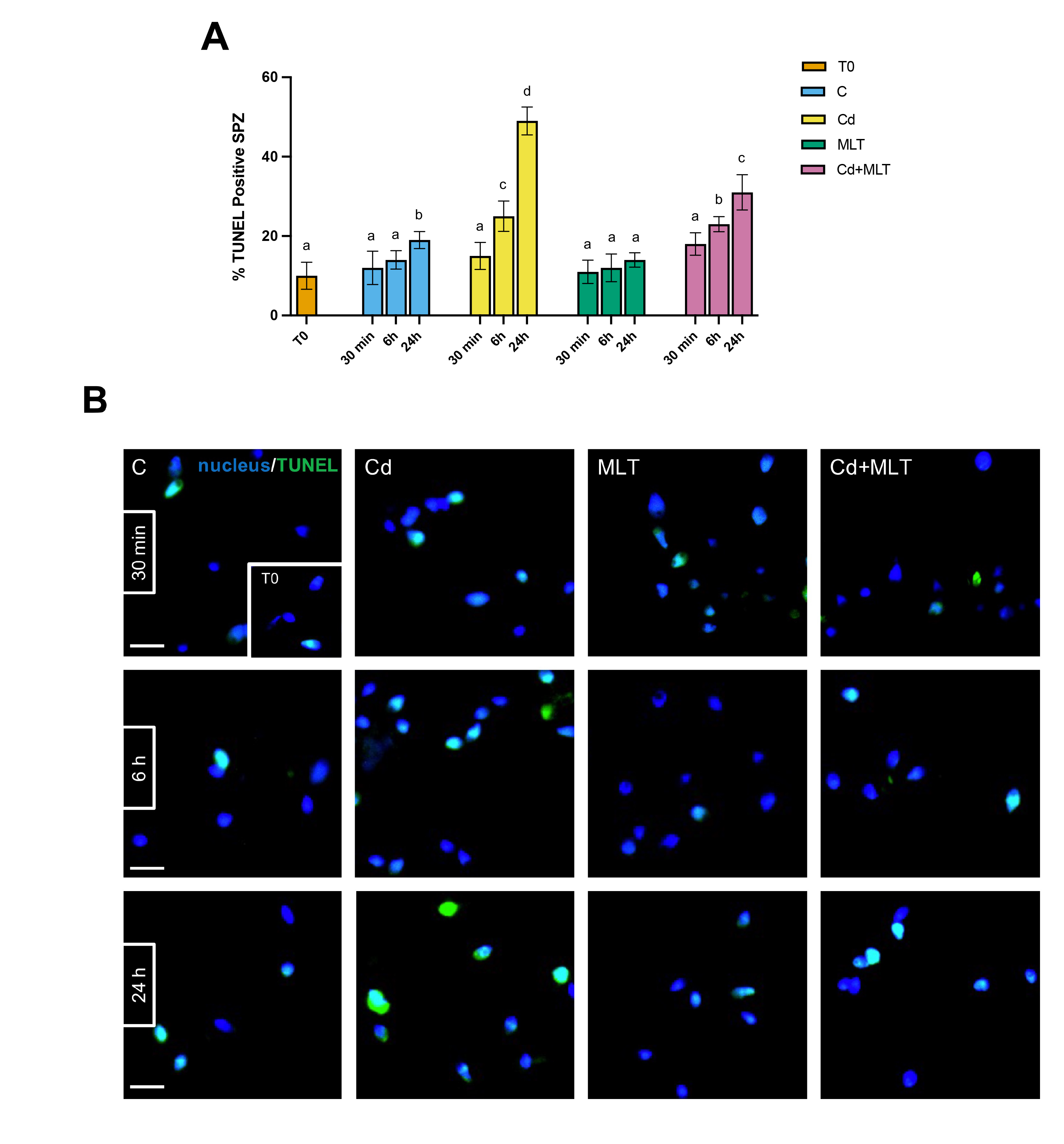

Supplement: Supplementary file 1 [file ijms-23-05128-s001.zip › Figure 3.tif]

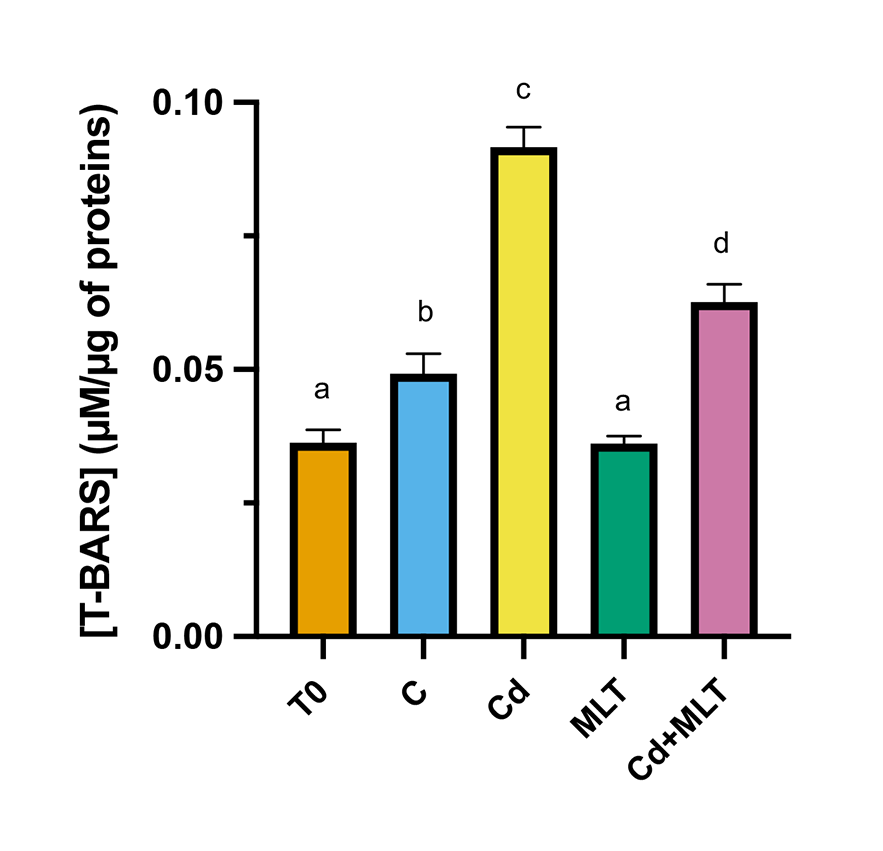

Supplement: Supplementary file 1 [file ijms-23-05128-s001.zip › Figure 4.tif]

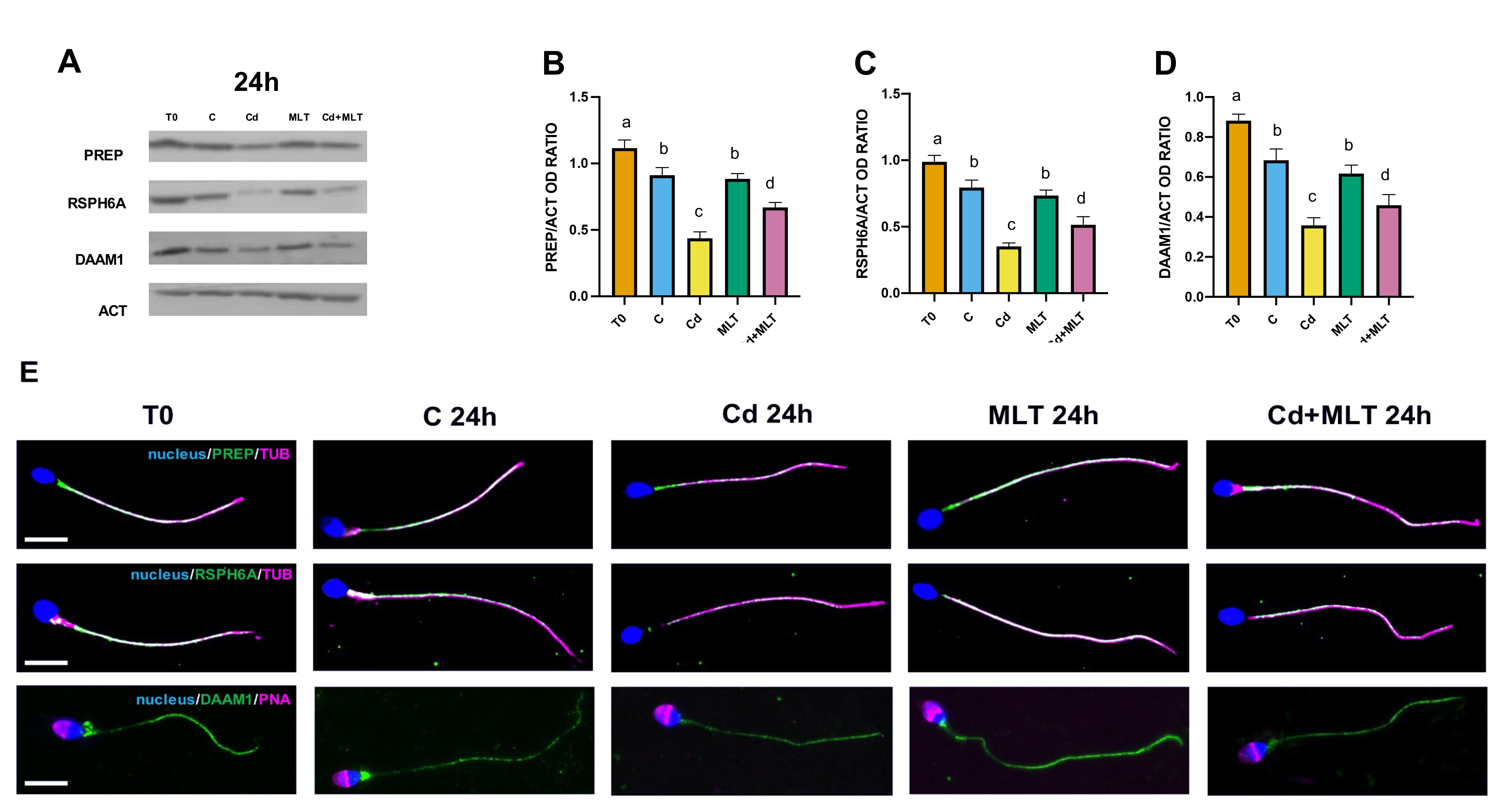

Supplement: Supplementary file 1 [file ijms-23-05128-s001.zip › Figure 5.tif]

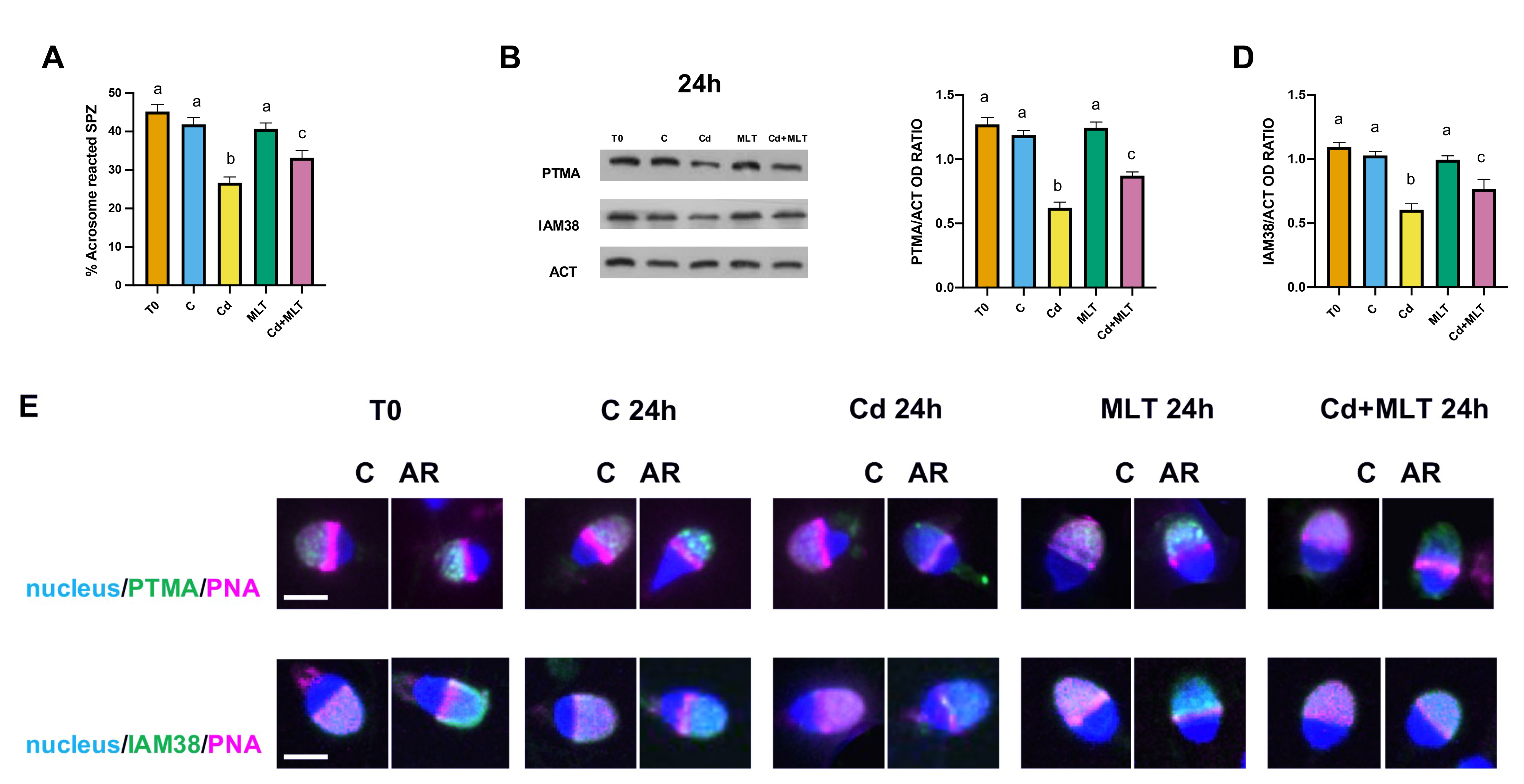

Supplement: Supplementary file 1 [file ijms-23-05128-s001.zip › Figure 6.tif]
